# Genomic information of the arsenic-resistant bacterium *Lysobacter arseniciresistens* type strain ZS79^T^ and comparison of *Lysobacter* draft genomes

**DOI:** 10.1186/s40793-015-0070-5

**Published:** 2015-10-27

**Authors:** Lin Liu, Shengzhe Zhang, Meizhong Luo, Gejiao Wang

**Affiliations:** State Key Laboratory of Agricultural Microbiology, College of Life Sciences and Technology, Huazhong Agricultural University, Wuhan, 430070 P. R. China

**Keywords:** *Lysobacter*, *Lysobacter arseniciresistens*, Comparative genomics, Genome sequence, *Xanthomonadaceae*

## Abstract

*Lysobacter arseniciresistens* ZS79^T^ is a highly arsenic-resistant,rod-shaped, motile, non-spore-forming, aerobic, Gram-negative bacterium. In this study, four *Lysobacter* type strains were sequenced and the genomic information of *L. arseniciresistens* ZS79^T^ and the comparative genomics results of the *Lysobacter* strains were described. The draft genome sequence of the strain ZS79^T^ consists of 3,086,721 bp and is distributed in 109 contigs. It has a G+C content of 69.5 % and contains 2,363 protein-coding genes including eight arsenic resistant genes.

**Electronic supplementary material:**

The online version of this article (doi:10.1186/s40793-015-0070-5) contains supplementary material, which is available to authorized users.

## Introduction

*Lysobacter arseniciresistens* type strain ZS79^T^ (=CGMCC 1.10752^T^ = KCTC 23365 T) belongs to family *Xanthomonadaceae* [1]. It is an arsenic-resistant bacterium isolated from subsurface soil of Tieshan iron mine, Daye City, P. R. China [1]. So far, there are 32 validly published species of *Lysobacter* [2]. Most of these *Lysobacter* strains were isolated from soil except that *Lysobacter brunescens* [3] and *Lysobacter oligotrophicus* [4] were isolated from water, and *Lysobacter concretionis* [5], *Lysobacter**daecheongensis* [6] *Lysobacter spongiicola* [7] were isolated from sludge, sediment and deep-sea sponge, respectively.

So far, the genomic sequences of two *Lysobacter* strains have been published (*Lysobacter capsici* AZ78 [8, 9] and *Lysobacter antibioticus* 13-6 [10]), but the annotation of *L. antibioticus* 13-6 was not completed. In order to provide genome information of genus *Lysobacter*, we performed whole genome sequencing of four strains of *Lysobacter* (*L. arseniciresistens* ZS79^T^, *Lysobacter**conceretionis* Ko07^T^ [5], *Lysobacter daejeonensis* GH1-9^T^ [11], and *Lysobacter defluvii* IMMIB APB-9^T^ [12]). In this study, the genome features of *L. arseniciresistens* ZS79^T^ is provided and the comparative results of five genomes of *Lysobacter* are presented.

## Organism information

### Classification and features

Members of genus *Lysobacter* are rod-shaped, aerobic, Gram-negative bacteria [3]. Their G+C contents are 65.4–70.1 %. They use NO_3_^−^, NH_4_^+^, glutamate, asparaginate as sole nitrogen sources, Q-8 as the major respiratory quinone, and diphosphatidylglycerol, phosphatidylethanolamine, phosphatidylglycerol, phosphatidyl-N-methylethanolamine as the major polar lipids [3, 8]. In addition, they could lyse cells of many creatures including bacteria, filamentous fungi, yeasts, algae and nematodes [3].

**Fig. 1:**
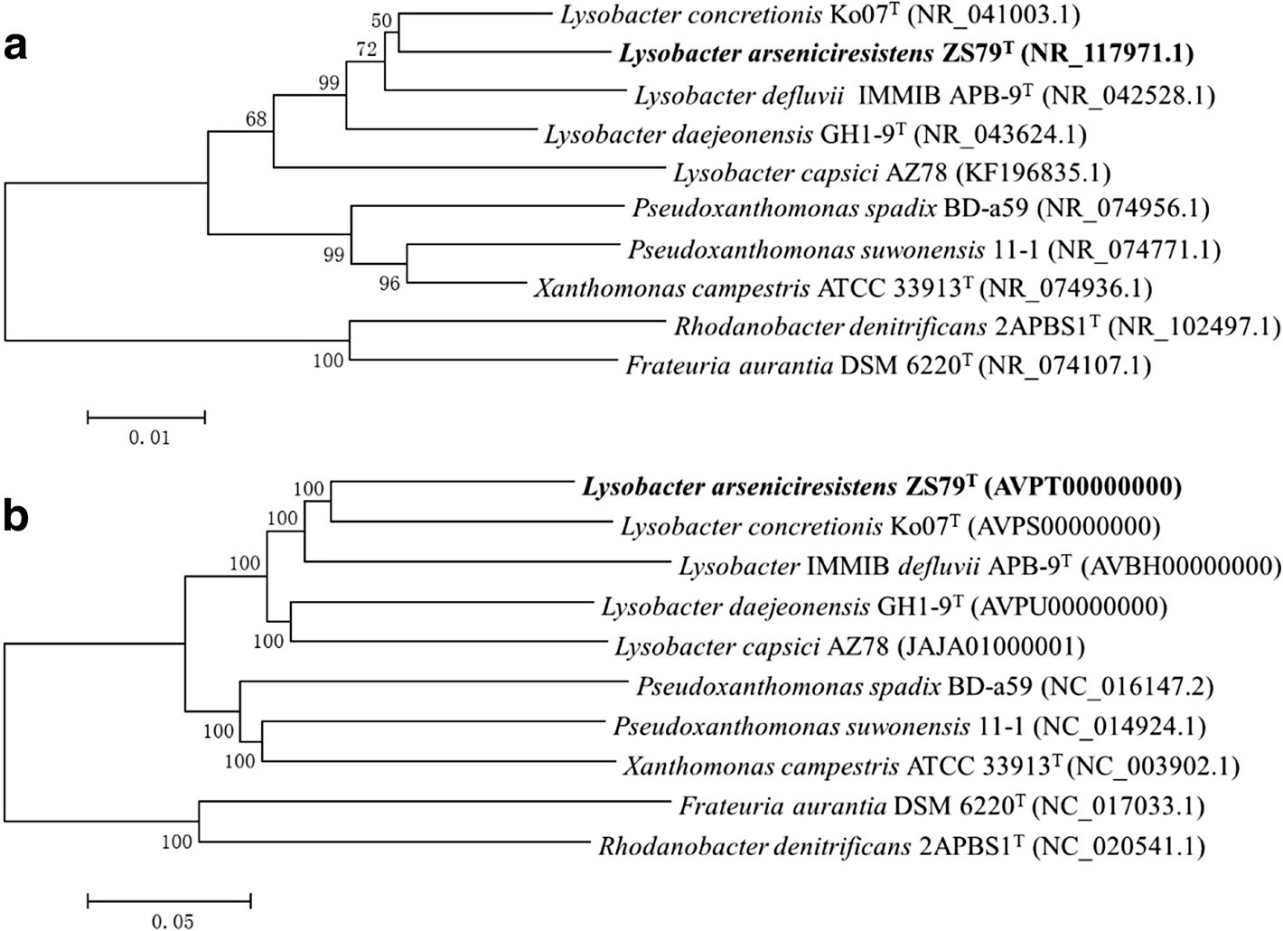
Phylogenetic analyses indicating the position of *L. arseniciresistens* (in bold) in family *Xanthomonadaceae*. **a** The NJ tree based on aligned sequences of 16S rRNA of ten strains of family *Xanthomonadaceae*. **b** The NJ tree based on 831 conserved proteins among the ten *Xanthomonadaceae* strains. Phylogenetic analyses were performed using MEGA version 6 [33]. The trees were built using p-distance model and a bootstrap analysis of 1000 replicates. The GenBank numbers are listed after each strain

Phylogenetic analyses of *L. arseniciresistens* ZS79^T^ and its related strains of family *Xanthomonadaceae* were performed based on 16S rRNA genes (Fig. 1a) and 831 conserved proteins (Fig. 1b). In both trees, strain ZS79^T^ is clustered with the other four strains of genus *Lysobacter*. The phylogenies of the two trees are similar but genomic based tree is more stable than the 16S rRNA gene one (Fig. 1b vs 1a).

**Fig. 2:**
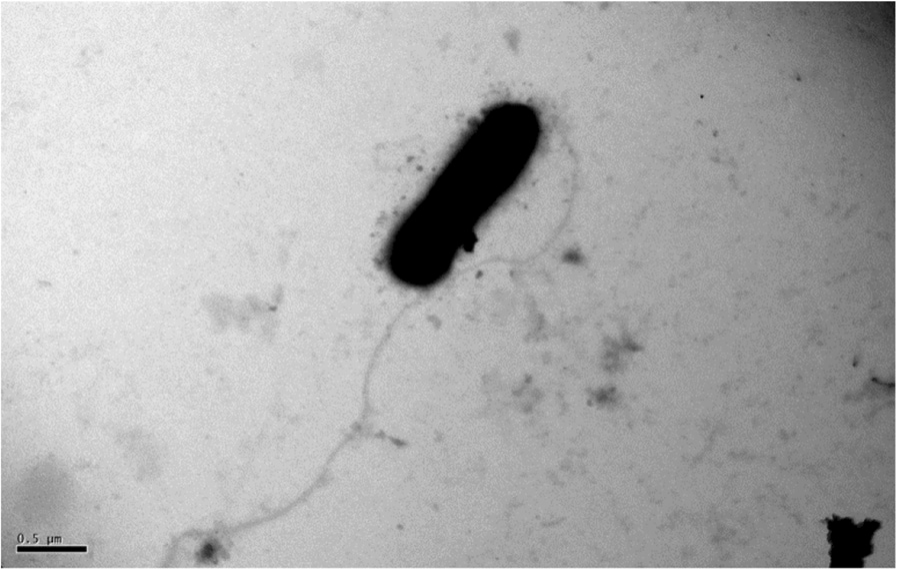
Transmission electron microscopy of *L. arseniciresistens* ZS79^T^

*L. arseniciresistens* ZS79^T^ is aerobic, motile, and Gram-negative bacterium with a Minimum Inhibitory Concentration of 14 mM arsenite in R2A medium (Table 1). The cells are rod-shaped with one flagellum and non-spore-forming (Fig. 2). Colonies of this strain are yellow, nontransparent, convex, circular, and, smooth [1].

**Table 1:** Classification and general features of *L. arseniciresistens* ZS79^T^ according to the MIGS recommendations [27]

MIGS ID	Property	Term	Evidence code^a^
	Classification	Domain *Bacteria*	TAS [28]
		Phylum *Proteobacteria*	TAS [29]
		Class *Gammaproteobacteria*	TAS [29, 30]
		Order *Xanthomonadales*	TAS [30, 31]
		Family *Xanthomonadaceae*	TAS [30, 31]
		Genus *Lysobacter*	TAS [3]
		Species *Lysobacter arseniciresistens*	TAS [1]
		Type strain: ZS79^T^ (=CGMCC 1.10752^T^ = KCTC 23365^T^).	
	Gram stain	negative	TAS [1]
	Cell shape	rod-shaped	TAS [1]
	Motility	motile	TAS [1]
	Sporulation	non-spore-forming	TAS [1]
	Temperature range	4–37 °C	TAS [1]
	Optimum temperature	28 °C	TAS [1]
	pH range; Optimum	5.0–9.0; 7.0	TAS [1]
	Carbon source	tyrosine, hippurate, gelatin, 3-hydroxybutyric acid	TAS [1]
MIGS-6	Habitat subsurface soil		TAS [1]
MIGS-6.3	Salinity	0–4 % NaCl (w/v)	TAS [1]
MIGS-22	Oxygen requirement	aerobic	TAS [1]
MIGS-15	Biotic relationship	free-living	NAS
MIGS-14	Pathogenicity	non-pathogen	NAS
MIGS-4	Geographic location	Daye City, Hubei province, China	TAS [1]
MIGS-5	Sample collection	2011	TAS [1]
MIGS-4.1	Latitude	30.207178 N	TAS [1]
MIGS-4.2	Longitude	114.901092 E	TAS [1]
MIGS-4.4	Altitude	not reported	

a: Evidence codes – TAS: Traceable Author Statement (i.e., a direct report exists in the literature); NAS: Non-traceable Author Statement (i.e., not directly observed for the living, isolated sample, but based on a generally accepted property for the species, or anecdotal evidence). These evidence codes are from the Gene Ontology project [32]

The major ubiquinone is Q-8, the major cellular fatty acids (>10 %) are iso-C_15__: 0_, iso-C_17__:1_*ω*9ϲ, iso-C_16__:0_, iso-C_11__:0_ and iso-C_11__:0_ 3-OH. The polar lipids are diphosphatidylglycerol, phosphatidylethanolamine, phosphatidylglycerol and a kind of unknown phospholipid The C + G content is was 70.7 mol% (HPLC) [1].

## Genome sequencing and annotation

### Genome project history

The genome of *L. arseniciresistens* ZS79^T^ was sequenced in April, 2013 and finished within two months. The high-quality draft genome sequence is available in GenBank database under accession number AVPT00000000. The genome sequencing project information is summarized in Table 2.

**Table 2:** Project information

MIGS ID	Property	Term
MIGS 31	Finishing quality	High-quality draft
MIGS-28	Libraries used	Illumina Paired-End library (300 bp insert size)
MIGS 29	Sequencing platforms	Illumina Hiseq2000
MIGS 31.2	Fold coverage	272.6×
MIGS 30	Assemblers	SOAPdenovo v1.05
MIGS 32	Gene calling method	GeneMarkS+
	Locus Tag	N799
	GenBank ID	AVPT00000000
	GenBank Date of Release	2014/10/24
	GOLD ID	Gi0055236
	BIOPROJECT	PRJNA214588
MIGS 31	Source Material Identifier	ZS79^T^
	Project relevance	Genome comparison

## Growth conditions and genomic DNA preparation

*L. arseniciresistens* ZS79^T^ was cultured in 50 ml of LB (Luria–Bertani) medium at 28 °C for 3 days with 160 160 r/min shaking. About 10 mg cells were harvested by centrifugation and suspended in normal saline, and then lysed using lysozyme. DNA was isolated using cells were harvested by centrifugation and suspended in normal saline, and then lysed using lysozyme. The DNA was extracted and purified using the QiAamp kit according to the manufacturer’s instruction (Qiagen, Germany).

## Genome sequencing and assembly

The whole genome sequencing of *L. arseniciresistens* ZS79^T^ was performed on Illumina Hiseq2000 with Paired-End library strategy (300 bp insert size) at Majorbio Biomedical Science and Technology Co. Ltd. DNA libraries with insert sizes from 300 to 500 bp was constructed using the established protocol [13]. The obtained high quality data contains 4,528,542 × 2 pared reads and 194,996 single reads with an average read length of 91 bp. The sequencing depth was 272.6×. Using SOAPdenovo v1.05 [14] the reads were assembled into 109 contigs with a cumulative genome size of 3,086,721 bp.

## Genome annotation

The draft sequence of *L. arseniciresistens* ZS79^T^ was annotated using the National Center for Biotechnology Information Prokaryotic Genomes Annotation Pipeline [15]. The functions of the predicted genes were determined through blast alignment against the NCBI protein database. Genes were identified using the gene caller GeneMarkS^+^ with the similarity-based gene detection approach [16]. The different features were predicted by WebMGA [17], TMHMM [18] and SignalP [19].

## Genome properties

**Fig. 3:**
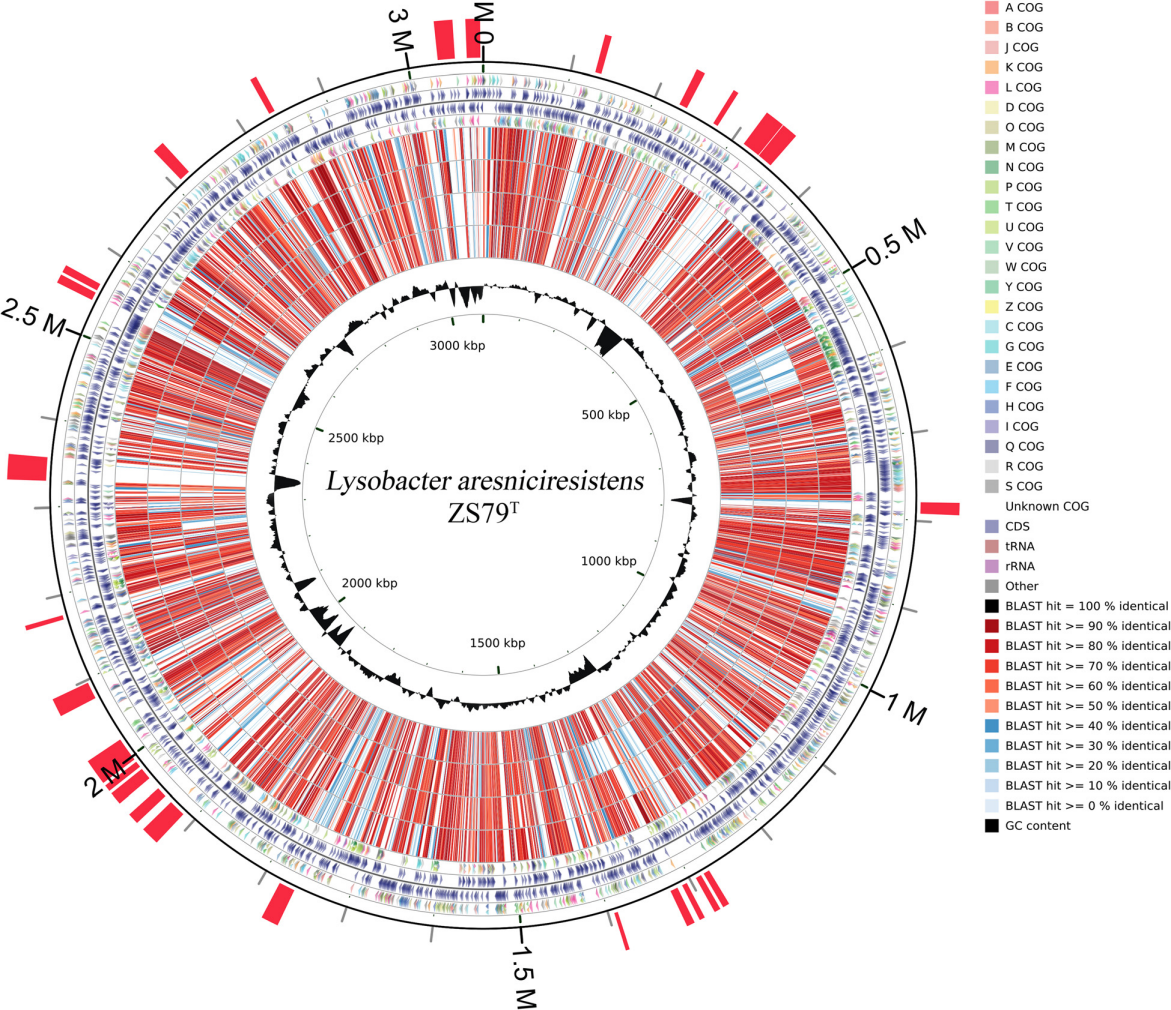
Graphical circular map of *L. arseniciresistens* ZS79^T^ genome. From outer to inner, ring 1 shows the genomic islands (red bars) that were predicted by IslandViewer [34]; ring 3,4 show the predicted genes on forward/reverse strand; ring 2,5 show the genes assigned to COGs; ring 6-9 show the ORFs similarity between the genome of *L. arseniciresistens* ZS79^T^ and the genomes of *L. conceretionis* Ko07^T^, *L. daejeonensis* GH1-9^T^, *L. capsici* AZ78 and *L. defluvii* IMMIB APB-9^T^; ring 10 shows the G+C% content plot

The whole genome sequence of *L. arseniciresistens* ZS79^T^ is 3,086,721 bp long with a G+C content of 69.6 % and is distributed into 109 contigs. It has 2,422 predicted genes including 2,363 (97.6 %) protein coding genes, 50 (2.1 %) RNA genes, and 9 (0.4 %) pseudo genes. A total of 1633 (67.4 %) genes have functional prediction, and 1,858 (76.7 %) genes could be assigned to Clusters of Orthologous Groups [20]. More detailed information of the genome statistics is showed in Table 3. The protein functional classification according to COGs is showed in Table 4. The genome map is showed in Fig. 3.

**Table 3:** Genome statistics

Attribute	Value	% of Total
Genome size (bp)	3,086,721	100.00
DNA coding (bp)	2,284,152	74.00
DNA G+C (bp)	2,147,191	69.56
DNA scaffolds	109	
Total genes	2,422	100.00
Protein coding genes	2,363	97.56
RNA genes	50	2.06
Pseudo genes	9	0.37
Genes in internal clusters	811	34.32
Genes with function prediction	1633	67.42
Genes assigned to COGs	1858	76.71
Genes with Pfam domains	2038	84.14
Genes with signal peptides	539	22.81
Genes with transmembrane helices	527	22.25
CRISPR repeats	1	0.41

**Table 4:** Number of genes associated with general COG functional categories

Code	Value	%age	Description
J	157	6.48	Translation, ribosomal structure and biogenesis
A	1	0.04	RNA processing and modification
K	116	4.79	Transcription
L	127	5.24	Replication, recombination and repair
B	2	0.08	Chromatin structure and dynamics
D	27	1.11	Cell cycle control, Cell division, chromosome partitioning
V	37	1.53	Defense mechanisms
T	104	4.29	Signal transduction mechanisms
M	125	5.16	Cell wall/membrane biogenesis
N	73	3.01	Cell motility
U	89	3.67	Intracellular trafficking and secretion
O	108	4.46	Posttranslational modification, protein turnover, chaperones
C	128	5.28	Energy production and conversion
G	70	2.89	Carbohydrate transport and metabolism
E	148	6.11	Amino acid transport and metabolism
F	50	2.06	Nucleotide transport and metabolism
H	91	3.76	Coenzyme transport and metabolism
I	90	3.72	Lipid transport and metabolism
P	107	4.42	Inorganic ion transport and metabolism
Q	53	2.19	Secondary metabolites biosynthesis, transport and catabolism
R	233	9.62	General function prediction only
S	185	7.64	Function unknown
-	564	23.29	Not in COGs

The total is based on the total number of protein coding genes in the genome

## Insights from the genome sequences

**Fig. 4:**
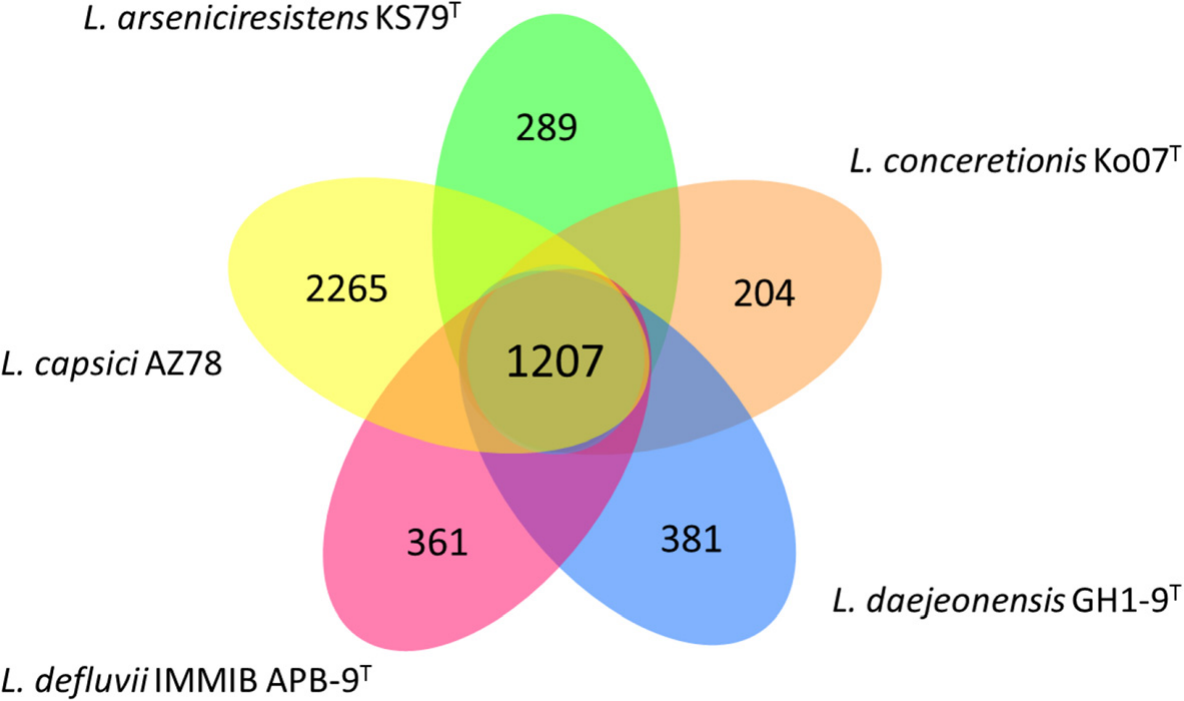
The core-genome and the unique genes of the five *Lysobacter* genomes. The Venn diagram shows the number of orthologous gene families of the core-genome (in the center) and the numbers of unique genes of each genome

To obtain features of *Lysobacter* genomes, we sequenced four genomes of genus *Lysobacter* and performed comparative genomic analysis among the five available genomes of this genus. The general features of these five genomes are summarized in Table 5. To calculate the pan-genome and core-genome of these five genomes, we performed orthologs clustering analysis using OrthoMCL [21]. The pan-genome has 6,409 orthologs families and the core-genome has 1,207 orthologs. The numbers of unique genes of each genome are showed in Fig. 4. To evaluate the genome variation of these five genomes, we first performed multiple alignments among these genome sequences using MAUVE [22] and then calculated the nucleotide diversity using DnaSP v5 [23]. These five genomes shared 0.73 Mb co-linear sequences. The π value of these sequences among these five genomes is 0.173 which means that the approximate nucleotide sequence homology is 83 % among genomes of *Lysobacter* [23].

**Table 5:** General features of the five *Lysobacter* genomes^a^

Strains	Source	Size (Mb)	G+C content	CDSs	rRNA clusters	tRNAs	Genome status			GenBank No.
							Draft/finished	Contigs	Contigs N50 (bp)	
*L. arseniciresistens* ZS79^T^	Iron-mined soil	3.1	69.58 %	2,363	3	46	Draft	109	101,761	AVPT00000000
*L. conceretionis* Ko07^T^	Anaerobic granules	3.0	67.25 %	2,232	3	46	Draft	26	386,139	AVPS00000000
*L. daejeonensis* GH1-9^T^	Green house soils	3.3	67.29 %	2,570	4	48	Draft	99	101,460	AVPU00000000
*L. defluvii* IMMIB APB-9^T^	Municipal solid waste	2.7	70.22 %	2,443	13	44	Draft	578	16,113	AVBH00000000
*L. capsici* AZ78	Tobacco & tomato rhizosphere	6.3	66.43 %	5,139	8	65	Draft	174	101,988	JAJA00000000

^a^The genome of *L. arseniciresistens* ZS79^T^, *L. conceretionis* Ko07^T^, *L. daejeonensis* GH1-9^T^ and *L. defluvii* IMMIB APB-9^T^ are sequenced in this study. The genome of *L. capsici* AZ78 was sequenced by Puoplo et al. [9]

In the genome of *L. arseniciresistens* ZS79^T^, we found that the genomic island distributions are consistent with the genome C + G content anomaly areas (Fig. 3). In addition, few gene sequences from the other four *Lysobacter* genomes could be aligned with these genomic island regions (Fig. 3, ring 6 to ring 9). These results indicated that the genes within the genomic islands were most probably acquired by horizontal transfer [24] and these regions are unique in the genome of *L. arseniciresistens* ZS79^T^.

According to Kyoto Encyclopedia of Genes and Genomes [25] annotation result, all of the five *Lysobacter* genomes have a nearly complete type II secretion system which could secret cell wall degrading enzymes [26]. This result may correspond to the behavior of *Lysobacter* members that were able to lyse cells of many microorganisms [3]. In addition, the genomes of *L. arseniciresistens* ZS79^T^, *L. concretionis* Ko07^T^ and *L. defluvii* IMMIB APB-9^T^ contain genes for flagellar assembly, whereas the genome of *L. daejeonensis* GH1-9^T^ does not contain any genes for flagellar assembly and *L. capsici* AZ78 does not contain genes for flagellar filament (Additional file 1: Table S2). These genotypes correspond to the phenotype descriptions that *L. daejeonensis* and *L. capsici* are non-motile [8, 11].

Genomic analysis showed eight genes corresponding to arsenic resistance in the genomes of *L. arseniciresistens* ZS79^T^ (Additional file 1: Table S3). This result well explained the arsenite resistance of this strain [1]. By contrast, fewer arsenic resistance were found in the genomes of *L. concretionis* Ko07^T^, *L. defluvii* IMMIB APB-9^T^, *L. capsici* AZ78, and *L. daejeonensis* GH1-9^T^ compared to strain ZS79^T^.

## Conclusions

The genomic information of *L. arseniciresistens* ZS79^T^ and the comparative genomics analysis of the five *Lysobacter* strains are obtained. The genomic based phylogeny is in agreement with the 16S rRNA gene based one indicating the usefulness of genomic information for bacterial taxonomic classification. Analysis of the genomes show certain correlation between the genotypes and the phenotypes.

## Additional file

Additional file 1:
**Table S1.** The proteins for Type II secretion in Lysobacter genomes. **Table S2.** The proteins for flagellar assembly in Lysobacter genomes. **Table S3.** The arsenic resistances genes found in five Lysobacter genomes. (XLSX 17 kb)

## References

[1] Luo G, Shi Z, Wang G (2012). *Lysobacter arseniciresistens* sp. nov., an arsenite-resistant bacterium isolated from iron-mined soil. Int J Syst Evol Microbiol.

[2] NCBI Taxonomy Browser http://www.ncbi.nlm.nih.gov/Taxonomy/Browser/wwwtax.cgi

[3] Christensen P, Cook FD (1978). *Lysobacter*, a New Genus of Nonfruiting, Gliding Bacteria with a High Base Ratio. Int J Syst Bacteriol.

[4] Fukuda W, Kimura T, Araki S, Miyoshi Y, Atomi H, Imanaka T (2013). *Lysobacter oligotrophicus* sp. nov., isolated from an Antarctic freshwater lake in Antarctica. Int J Syst Evol Microbiol.

[5] Bae HS, Im WT, Lee ST (2005). *Lysobacter concretionis* sp. nov., isolated from anaerobic granules in an upflow anaerobic sludge blanket reactor. Int J Syst Evol Microbiol.

[6] Ten LN, Jung HM, Im WT, Yoo SA, Lee ST (2008). *Lysobacter daecheongensis* sp. nov., isolated from sediment of stream near the Daechung dam in South Korea. J Microbiol.

[7] Romanenko LA, Uchino M, Tanaka N, Frolova GM, Mikhailov VV (2008). *Lysobacter spongiicola* sp. nov., isolated from a deep-sea sponge. Int J Syst Evol Microbiol.

[8] Park JH, Kim R, Aslam Z, Jeon CO, Chung YR (2008). *Lysobacter capsici* sp. nov., with antimicrobial activity, isolated from the rhizosphere of pepper, and emended description of the genus *Lysobacter*. Int J Syst Evol Microbiol.

[9] Puopolo G, Sonego P, Engelen K, Pertot I. Draft Genome Sequence of *Lysobacter capsici* AZ78, a Bacterium Antagonistic to Plant-Pathogenic Oomycetes. Genome Announc. 2014;2. PubMed http://www.ncbi.nlm.nih.gov/pubmed/24762937. 10.1128/genomeA.00325-14 PMC3999494 24762937

[10] Zhou L, Li M, Yang J, Wei L, Ji G. Draft Genome Sequence of Antagonistic Agent *Lysobacter antibioticus* 13-6. Genome Announc. 2014;2. PubMed http://www.ncbi.nlm.nih.gov/pubmed/25301638. 10.1128/genomeA.00566-14 PMC4192370 25301638

[11] Weon HY, Kim BY, Baek YK, Yoo SH, Kwon SW, Stackebrandt E (2006). Two novel species, *Lysobacter daejeonensis* sp. nov. and *Lysobacter yangpyeongensis* sp. nov., isolated from Korean greenhouse soils. Int J Syst Evol Microbiol.

[12] Yassin AF, Chen WM, Hupfer H, Siering C, Kroppenstedt RM, Arun AB (2007). *Lysobacter defluvii* sp. nov., isolated from municipal solid waste. Int J Syst Evol Microbiol.

[13] Illumina official website http://www.illumina.com

[14] Luo R, Liu B, Xie Y, Li Z, Huang W, Yuan J (2012). SOAPdenovo2: an empirically improved memory-efficient short-read de novo assembler. Gigascience.

[15] Prokaryotic Genome Annotation Pipeline http://www.ncbi.nlm.nih.gov/genome/annotation_prok.

[16] Besemer J, Lomsadze A, Borodovsky M (2001). GeneMarkS: a self-training method for prediction of gene starts in microbial genomes. Implications for finding sequence motifs in regulatory regions. Nucleic Acids Res.

[17] Wu S, Zhu Z, Fu L, Niu B, Li W (2011). WebMGA: a customizable web server for fast metagenomic sequence analysis. BMC Genomics.

[18] Krogh A, Larsson BÈ, Von Heijne G (2001). Predicting transmembrane protein topology with a hidden Markov model: application to complete genomes. J Mol Biol.

[19] Dyrlov Bendtsen J, Nielsen H, von Heijne G (2004). Improved prediction of signal peptides: SignalP 3.0. J Mol Biol.

[20] Tatusov RL, Fedorova ND, Jackson JD, Jacobs AR, Kiryutin B, Koonin EV (2003). The COG database: an updated version includes eukaryotes. BMC Bioinformatics.

[21] Li L, Stoeckert CJ, Roos DS (2003). OrthoMCL: identification of ortholog groups for eukaryotic genomes. Genome Res.

[22] Darling AC, Mau B, Blattner FR, Perna NT (2004). Mauve: multiple alignment of conserved genomic sequence with rearrangements. Genome Res.

[23] Librado P, Rozas J (2009). DnaSP v5: a software for comprehensive analysis of DNA polymorphism data. Bioinformatics.

[24] Langille MG, Hsiao WW, Brinkman FS (2010). Detecting genomic islands using bioinformatics approaches. Nat Rev Microbiol.

[25] Kanehisa M, Goto S, Sato Y, Kawashima M, Furumichi M, Tanabe M (2014). Data, information, knowledge and principle: back to metabolism in KEGG. Nucleic Acids Res.

[26] Cianciotto NP (2005). Type II secretion: a protein secretion system for all seasons. Trends Microbiol.

[27] Field D, Garrity G, Gray T, Morrison N, Selengut J, Sterk P (2008). The minimum information about a genome sequence (MIGS) specification. Nat Biotechnol.

[28] Woese CR, Kandler O, Wheelis ML (1990). Towards a natural system of organisms: proposal for the domains Archaea, Bacteria, and Eucarya. Proc Natl Acad Sci U S A.

[29] Garrity G, Bell J, Lilburn T, Garrity G, Brenner D, Krieg N, Staley J (2005). Phylum XIV. *Proteobacteria* phyl. nov. Bergey’s Manual of Systematic Bacteriology.

[30] Validation of publication of new names and new combinations previously effectively published outside the IJSEM. Int J Syst Evol Microbiol. 2005; 55:1743–5. PubMed [http://www.ncbi.nlm.nih.gov/pubmed/16166658]. 10.1099/ijs.0.63996-0 16166658

[31] Saddler G, Bradbury J, Garrity G, Brenner D, Krieg N, Staley J (2005). Order III. *Xanthomonadales* ord. nov. Bergey’s Manual of Systematic Bacteriology.

[32] Ashburner M, Ball CA, Blake JA, Botstein D, Butler H, Cherry JM (2000). Gene ontology: tool for the unification of biology. The Gene Ontology Consortium. Nat Genet.

[33] Tamura K, Stecher G, Peterson D, Filipski A, Kumar S (2013). MEGA6: Molecular Evolutionary Genetics Analysis version 6.0. Mol Biol Evol.

[34] Langille MG, Brinkman FS (2009). IslandViewer: an integrated interface for computational identification and visualization of genomic islands. Bioinformatics.

